# Psychosocial care responses to terrorist attacks: a country case study of Norway, France and Belgium

**DOI:** 10.1186/s12913-022-07691-2

**Published:** 2022-03-24

**Authors:** Lise Eilin Stene, Cécile Vuillermoz, Roel Van Overmeire, Johan Bilsen, Michel Dückers, Lisa Govasli Nilsen, Stéphanie Vandentorren

**Affiliations:** 1 Norwegian Centre for Violence and Traumatic Stress Studies (NKVTS), Oslo, Norway; 2 Sorbonne Université, INSERM, Institut Pierre Louis d’épidémiologie et de Santé Publique (IPLESP), Department of social epidemiology, Paris, France; 3 Mental Health & Wellbeing research group, Vrije Universiteit Brussel, Brussels, Belgium; 4 ARQ National Psychotrauma Centre, Diemen, Netherlands; 5 Netherlands Institute of Health Services Research (NIVEL), Utrecht, Netherlands; 6 Faculty of Behavioural and Social Sciences, University of Groningen, Groningen, Netherlands; 7 Department of Sociology and Political Science, Norwegian University of Science and Technology, NTNU, Trondheim, Norway; 8 Santé publique France, Saint Maurice, France; 9 Université Bordeaux, Inserm, UMR 1219, Vintage team, Bordeaux, France

**Keywords:** Terrorism, Mass casualty incidents, Crisis intervention, Emergencies, Psychological trauma, Psychosocial interventions, Health services research, Mental health services, Program evaluation, Europe

## Abstract

**Background:**

The international terrorism threat urges societies to invest in the planning and organization of psychosocial care. With the aim to contribute to cross-national learning, this study describes the content, target populations and providers of psychosocial care to civilians after terrorist attacks in Norway, France and Belgium.

**Methods:**

We identified and reviewed pre- and post-attack policy documents, guidelines, reports and other relevant grey literature addressing the psychosocial care response to terrorist attacks in Oslo/Utøya, Norway on 22 July 2011; in Paris, France on 13 November 2015; and in Brussels, Belgium on 22 March 2016.

**Results:**

In Norway, there was a primary care based approach with multidisciplinary crisis teams in the local municipalities. In response to the terrorist attacks, there were proactive follow-up programs within primary care and occupational health services with screenings of target groups throughout a year. In France, there was a national network of specialized emergency psychosocial units primarily consisting of psychiatrists, psychologists and psychiatric nurses organized by the regional health agencies. They provided psychological support the first month including guidance for long-term healthcare, but there were no systematic screening programs after the acute phase. In Belgium, there were psychosocial intervention networks in the local municipalities, yet the acute psychosocial care was coordinated at a federal level. A reception centre was organized to provide acute psychosocial care, but there were no reported public long-term psychosocial care initiatives in response to the attacks.

**Conclusions:**

Psychosocial care responses, especially long-term follow-up activities, differed substantially between countries. Models for registration of affected individuals, monitoring of their health and continuous evaluation of countries’ psychosocial care provision incorporated in international guidelines may strengthen public health responses to mass-casualty incidents.

**Supplementary Information:**

The online version contains supplementary material available at 10.1186/s12913-022-07691-2.

## Background

Terrorist attacks cause a significant number of casualties worldwide and seek to create fear in the public [1, 2]. Their impact is widespread, affecting those directly exposed and their close ones, but also first responders, local communities and even the society at large. Acute stress reactions are common after exposure to such events, yet they often recede spontaneously within a month [3]. Still, some affected people develop long-term mental or physical health problems, or impaired functioning at work, school or in social relationships [4–12]. The prevalence of long-term health problems may differ by severity of exposure and individual risk factors but is also related to access to healthcare and psychosocial support. Unmet healthcare needs have indeed been observed after terrorist attacks, both among exposed individuals and in the general population [13–15].

Planning of psychosocial care in advance is important to efficiently respond to and recover from mass casualty incidents such as terrorist attacks [16]. Their unpredictability and the urgency of response make it challenging to organize appropriate and timely care and to identify people who need psychosocial care interventions. The chaotic circumstances also make it difficult to assess the efficiency of the implemented psychosocial care response and to develop scientific evidence on the best practices. International guidelines on psychosocial care after disasters are largely based on consensus of expert opinions and the available research, which is still scarce [17–24]. It has been recommended to promote natural recovery and to identify individuals at risk of developing posttraumatic health problems to assure that they receive treatment if needed [25]. Although there is limited evidence, research suggests that using a stepped care model with screening and triage might be a beneficial approach to provide psychosocial care after disasters [17, 26]. Through active monitoring of individuals at risk of developing mental health problems, one aims to be able to provide timely treatment to those who need it the most. However, in practice it is often challenging to identify and reach the target population(s) [27, 28]. A large number of people may be affected by an attack, including those present at the site of the attack when it occurred, professional or volunteer first responders, people living or working nearby, family members or friends of the survivors and the bereaved. The denotation of a target population for psychosocial care interventions may also depend on several factors: the quality of the registration by different agencies and service providers in the hours and days after the attack, legal issues, privacy concerns or the decisions of the stakeholders that are responsible for the planning and delivery of psychosocial care [27]. It also depends on the accessibility and quality of the existing mental health system and its capacity to accommodate a potential large flux of patients, as well as investments in a coordinated multi-agency psychosocial care planning and delivery capacity in the preparedness phase [29]. Ideally, disaster plans based on evidence-based guidelines are prepared in advance, providing a framework for a coordinated psychosocial care response that is regularly updated [17, 22, 29, 30]. Next, if a terrorist attack strikes, the ensuing psychosocial care response should be adapted to the specific event and the affected populations.

Although international guidelines have been developed, little is known about how different countries actually meet psychosocial care needs after terrorist attacks. Such knowledge is essential to strengthen the public health preparedness and response to such disasters across countries. Given the scarcity of evidence on the best practices for post-disaster psychosocial care, it is particularly important to accumulate experiences and documentation of practices and interventions that have been applied. This study was conducted after a public health workshop on healthcare after large-scale terrorist attacks in Norway, France and Belgium, and sheds light on these countries’ psychosocial care responses [31]. Our overall aim was to strengthen the knowledge base for future planning, implementation and evaluation of psychosocial care responses to terrorist attacks and similar disasters. More specifically, the objectives were to describe the documented content, target populations and providers of psychosocial care to civilians after these terrorist attacks in Norway, France and Belgium. Furthermore, we wanted to investigate how characteristics of the attacks and the countries’ health systems may have influenced the psychosocial care responses.

## Methods

### Study design and scope

This is a country case study of the public authorities’ psychosocial care responses to terrorist attacks in Norway, France and Belgium. The scope was on acute and long-term psychosocial care for the civilian population as outlined on a national level by official bodies in these countries. We focused on the attacks that caused the largest number of deaths in each country in the past decade: the 22 July 2011 attacks in Oslo and Utøya, Norway; the 13 November 2015 attacks in Paris, France; and the 22 March 2016 attacks in Brussels, Belgium. These attacks caused multiple deaths and injuries and exposed thousands to a potentially traumatic event. We reviewed pre-attack plans and guidelines for psychosocial care responses to mass casualty incidents, and post-attack documentation of the actual organization and content of psychosocial care in response to the terrorist attacks under study. The nature and availability of such documentation were unknown beforehand. Consequently, this study is exploratory and descriptive. Data were collected and examined by two researchers from each of the three included countries, next synthesized by all researchers and an additional Dutch researcher with expertise in international guideline development on postdisaster psychosocial care and cross-national comparison of the quality of psychosocial support. The researchers had multi-disciplinary backgrounds in medicine, nursing, epidemiology, public health, mental health, political science, sociology and public administration.

### Data material

The study included documents written in the national languages French, Dutch and Norwegian, and in English.

#### Psychosocial care responses

Psychosocial care is a broad term ranging from immediate comfort and practical help through long-term psychological support and specialist trauma care [32]. We searched for information about the content, target populations and providers of acute and long-term psychosocial care to the civilian population in response to the attacks in each country. Information on the nature and range of such applied public health interventions, about how, by whom and for whom they were implemented, the rationale for the approaches chosen, and whether changes were made, may be primarily or entirely held in grey literature [33]. Hence, we analyzed grey literature, such as governmental policy reports, national guidelines, and reports addressing the plans for a psychosocial care response to terrorist attacks or other mass casualty incidents, that were available when the terrorist attacks under study occurred, and post-attack documentation of the actual organization and content of psychosocial care in response to the attacks. Such information is typically not available in scientific literature or scientific databases. To identify relevant grey literature, we used a snowballing approach and searched the web sites of targeted ministries, governmental organizations and other relevant stakeholders in the respective countries. Next, we contacted professionals of the health authorities in each country who had been involved in the health response to the attacks. They advised us by e-mail regarding relevant documents concerning the attacks. Further, we also included articles from scientific journals describing the psychosocial care response that were authored by representatives from the health authorities or professionals who had provided psychosocial care in the wake of the attacks. Additional file 1 lists the documents and web sites we reviewed on the psychosocial care responses. The researchers from each country were asked to collect data to respond to the questions in Table 1. In case of conflicting information from different sources, documents commissioned with a formal mandate from the government or governmental institutions were prioritized.

**Table 1:** Questions to guide the data collection from each country

***I) Pre-attack:*** *Identify the most updated guidelines/plans/recommendations/documents available when the attacks under study occurred, describing the provision of psychosocial care after terrorist attacks or disasters in general. Describe the content of the psychosocial care regarding* *- the acute aftermath (first hours/days).* *- the medium- and long-term aftermath (weeks/months/years after the incident).* *Provide descriptions that are as close as possible to the formulations used in the documents.* ***II) Post-attack:*** *Identify the guidelines/plans/recommendations/documents describing the provision of psychosocial care in response to the attacks under study. Describe the content of the psychosocial care regarding* *- the acute aftermath (first hours/days).* *- the medium- and long-term aftermath (weeks/months/years after the incident).* *Describe if there were target populations for psychosocial care interventions, and if different types of care were offered for specific groups.* *Provide descriptions that are as close as possible to the formulations used in the documents.* ***III) Pre- and post-attack:*** *- Who were to be offered psychosocial care?* *- Did the planned psychosocial care include screening assessments?* *- If yes, when were the screenings to be performed?* *- If the psychosocial care included screenings, for whom were they to be performed?* *- Which healthcare providers/services were to provide psychosocial care?* *- What were their intended roles/tasks and how were they coordinated?* *Report also if there was other relevant information about the content and/or organization of psychosocial care that was not covered by the points above.*	

Our focus was on the public authorities’ psychosocial care responses to the attacks and how they documented what was done, by whom and for whom. We did not investigate the treatment of injuries, the emergency medical response beyond psychosocial care, legal aid or financial compensations. Since we did not aim to assess the achievements of the different psychosocial care responses, we did not include observational or qualitative research evaluating the health service utilization after the attacks. Furthermore, we did not examine psychosocial care to professional first responders who intervened in the attacks because of their profession, e.g. from the police, the fire brigade and the military, or medical personnel. The psychosocial care to professional first responders may be outlined in their respective specific institutional crisis plans rather than in general public health plans and may vary according to type of responder. Hence, it was considered too broad to be covered in this study. Those who were struck by the terrorist attacks while they were at work, such as airport personnel in Belgium and Ministry employees in Norway, were considered as civilians.

#### Characteristics of the attacks and the health systems

We collected information about certain characteristics of the attacks, such as the total number of fatalities (except perpetrators), fatalities in children (< 18 years old), the reported number of physically injured, the type of attack and location. If this information was not available in the documents listed in (Additional file 1), we used internet search engines such as Google to examine if this information was available in, e.g., articles in Wikipedia or renowned news media. Finally, we retrieved information on characteristics of the health systems, such as expenditure funded by public sources, role of general practitioners, and gatekeeping from country-based reports from, e.g., the Organisation for Economic Co-operation and Development (OECD) and the European Observatory on Health Systems and Policies.

### Ethics

This study was based on publicly available documents. There was no collection or storage of sensitive personal data. It did not involve human participants and did not require consent.

## Results

Table 2 presents characteristics of the attacks and the health system in each country.

**Table 2:** Characteristics of the terrorist attacks and the health systems in Norway, France and Belgium. References are reported in the results

	Norway	France	Belgium
**Characteristics of the terrorist attacks**	**Oslo and Utøya attacks 22 July 2011**	**Paris attacks 13 November 2015**	**Brussels attacks 22 March 2016**
Total number of fatalities (except perpetrators)	77	130	32
Number of fatalities in children (< 18 years old)	33	1	0
Reported number of physically injured^a^	172	493	340
Type of attack(s) and location(s)	- Bombing at government quarter in city centre (8 deaths).	- Suicide bombings outside football stadium (1 death).	- Two suicide bombings at airport (12 deaths).
	- Shooting at youth Labor party camp on small island (69 deaths).	- Hostage, shooting and suicide bombings at theatre concert (90 deaths).	- One suicide bombing at metro station in city centre (20 deaths).
		- Shootings and suicide bombings at bars/restaurants in four locations (39 deaths).	
**Characteristics of the health systems**			
Expenditure funded by public sources	85%	77%	77%
General practitioners (GPs) and gatekeeping of specialized mental health services	Gatekeeping system: The GPs are important providers of mental care and refer patients to specialized care when necessary. If patients consult a psychiatrist or psychologist without referral, they must pay full fees. Since a reform in 2001, over 99% of the population had a regular GP.	Semi-gatekeeping system: Provides incentives to consult a regular GP before a specialist. Patients who consult a psychiatrist without referral must pay a larger part of but not the entire fees. A study in 2007 indicated that 83% of the population had a regular GP.	No gatekeeping system: GPs do not serve as gatekeepers. Incentives have been made to promote their role in healthcare, e.g. increased reimbursement for first visit to a psychiatrist, and only reimbursement of psychologist consultation if referred by a GP or other physician. In a national health survey in 2008, almost 95% reported having a regular GP.
Main responsibility of organizing post-disaster psychosocial care	Local municipalities	Regional health agencies	Split responsibility: Federal authorities in the acute and local authorities in the post-acute phase

^a^Discrepant numbers reported in different sources. It is often unclear how physical injury has been defined and measured. Therefore, the numbers may not be comparable across attacks/countries

### Characteristics of the attacks

There were between 32 and 130 fatalities, excluding the deaths of the perpetrators [34–36]. The number of physically injured reported by the authorities ranged between 172 and 493 (Table 2) [37–39]. All the attacks took place at more than one location. The 2011 Norway attacks comprised both an urban and a rural attack site. The Labour party and its members were designated targets of the attacks [40]. Many of the victims were youth; 33 of the 77 fatalities were adolescents under the age of 18 years [36]. One person of non-Norwegian nationality was killed [41]. There was no recent history of terrorist attacks in Norway before the 2011 attacks. The November 2015 attacks in France was a multi-site attack in Paris and its suburb. Excluding the deaths of the perpetrators, 130 persons were killed in the attacks, including one under the age of 18 years and 24 of non-French nationality [34]. These attacks occurred 10 months after multi-site terrorist attacks in the same area [8]. Moreover, several terrorist attacks took place in France in its aftermath, including a large-scale attack in Nice 7 months after [42]. The 2016 attacks in Belgium comprised two suicide bombings at the Brussels airport and one in the metro in the city centre. The rescue services recorded that 340 persons were injured in the Brussels attacks [43]. In total, 18 of the 32 who were killed in the attacks were of non-Belgian nationality [35]. No children were killed in these attacks [44]. Brussels had also been struck by a terrorist attack in May 2014, i.e., approximately 2 years before [35].

### Characteristics of the health systems

All three countries under study had well developed health systems with nearly universal health coverage where most of the healthcare fees were publicly funded (Table 2) [45]. A difference in the countries’ organization of healthcare lay within primary care and the role of the General Practitioner (GP). In Norway, over 99% of the population had a regular GP [46]. The GP was both an important provider and coordinator of different types of care and served as gatekeeper for further referral to specialist care [47]. In France, there was a semi-gatekeeping system where patients were encouraged to access specialized healthcare through referral from a regular GP through financial incentives [48]. A study revealed that 83% of the population in France had a regular GP in 2007 [49]. In Belgium, there was no gatekeeping system, and the specialist, such as a psychiatrist, may form the first point of contact with the patient [50, 51]. However, reimbursements of some healthcare workers (e.g., psychologists in ambulatory centers) are only possible when patients are referred by a physician [52]. Almost 95% of the respondents of a national health survey conducted in 2008 reported having a regular GP [53]. As for the organization of post-disaster psychosocial care to civilians, this was primarily the responsibility of the local municipalities in Norway [54] and of the regional health services in France [55]. In Belgium there was a split responsibility, where the federal authorities were first responsible for the acute psychosocial care, and later it was transferred to the local communities in the post-acute phase [56].

### The outlined psychosocial care responses

Tables 3, 4, 5 summarize the information we identified about the content, target populations, providers and timing of the psychosocial care interventions in each country after the attacks under study. The documentation we examined presented a variable range of formats, level of details and subject matter across countries. Consequently, we introduce the results from each country with a short description of the nature and levels of details of the documentation of the psychosocial care responses.

**Table 3:** Information identified about the psychosocial care response, Oslo and Utøya 22 July 2011 attacks, Norway. Acute = first hours/days after attacks. Medium/long-term = weeks/months/years after the attacks

Timing	Target population	Providers	Description of psychosocial care	References
Acute	Anyone affected by the attacks.	Municipal primary care based multidisciplinary crisis teams. In Oslo, there was a multidisciplinary standby crisis service at the out-of-hours primary care centre that could alert other personnel if needed, e.g., from psychiatric clinics. Ambulance crew and health personnel at the attack sites.	Municipal multidisciplinary crisis teams across the country provided acute psychosocial care to victims of the attacks and their relatives/close ones, as the survivors, their families and the bereaved lived geographically spread in all regions of Norway. Immediate support for psychosocial shock reactions and at-site crisis response based on Hobfoll’s principles of psychological first aid (sense of safety, calming, sense of self- and community efficacy, connectedness, hope).	[21, 38, 54]
Acute	Ministerial employees affected by the bomb and their relatives. Overall 310 were at work. In total, there were around 3500 employees.	Occupational health services of the Ministries with specialist support.	A drop-in crisis centre for ministerial employees and their relatives was set up at a hotel nearby the site of the bombing, where they were offered defusing to alleviate acute stress as well as support in groups. Information meetings were arranged.	[38, 57]
Acute	Survivors of the Utøya youth camp attack (495 survivors, mostly adolescents and young adults) and their families, and families of the 69 persons killed.	Primary care crisis team of affected municipality composed of a medical officer for health (MOH), a chaplain and staff from the social services, with assistance from crisis team in neighbor municipality, personnel from nearby psychiatric clinics and paramedics. At the provisional crisis centre there was access to medical doctors, psychiatrists, psychologists, nurses, a chaplain and imam.	Acute psychosocial care and psychological first aid. In the first hours, the MOH, who was also a regular GP in the affected municipality, had coordinator responsibility and requisitioned a hotel nearby the Utøya island as a crisis centre. A more comprehensive psychosocial emergency response was organized from 02 a.m. on 23 July (around 7 h after the shooting ended), comprising sessions with group counselling, individual counselling, information meetings and health checks at this provisional crisis centre. It was open until 1 p.m. on July 26	[38, 58]
Acute	Relatives of the severely injured.	Multidisciplinary psychosocial crisis team at Oslo University Hospital (OUH) Ullevål composed of adult and child & adolescent psychiatrists, psychologists, nurses, social workers, clerical staff and a chaplain.	Separate crisis centres were established for relatives of survivors and relatives of the deceased/missing persons to provide acute psychosocial care and information.	[38]
Acute	Relatives of missing persons and the deceased.	Multidisciplinary psychosocial crisis team at OUH Rikshospitalet. In addition, there was a police-run crisis centre which was initially at a police station, next moved to a hotel in city centre the day after the attacks.	See above. OUH Rikshospitalet is at a different location in Oslo than Ullevål.	[38]
Acute/ Medium/ long-term	Non-organised voluntary helpers in boats or at the Utøya camp site.	Team composed of a psychiatrist, psychiatric nurse and public health nurse dispatched to the camp site the first 2 days following the attacks. Next, there was a drop-in arrangement at the council premises in Hole municipality attended by a team of health personnel and group sessions led by a psychiatrist and a clinical social worker.	Meetings were arranged at the camp site café the 2 days following the attacks. Over the following 3 weeks, there was a drop-in arrangement at the council premises in Hole municipality for all volunteers. A week after the Utøya attack, the head of the local municipality’s crisis team (clinical social worker) set up groups for regular follow-up in conjunction with the head of a close-by psychiatric centre (psychiatrist). Weekly sessions were held for approx. 20–30 participants at a time. This follow-up was originally planned through the first 3 months after the attacks, but the group wished to continue with monthly sessions.	[38]
Medium/ long-term	Anyone affected by the attacks.	Municipal multidisciplinary crisis teams, regular GPs, specialized mental health services.	A general principle of using the lowest effective level of care. Principles of psychological first aid were to be pursued as well as facilitation of controlled re-exposure. Watchful waiting as described in the NICE guidelines (i.e., regularly monitoring persons with some symptoms not (yet) receiving active interventions). If needed, referral to specialized treatment by regular GP. Trauma-focused Cognitive Behavioral Therapy (TF-CBT) or Eye Movement Desensitization Reprocessing (EMDR) were recommended if there was a need for specialized treatment of PTSD.	[38, 54]
Medium/long-term	Ministerial employees affected by the bomb in the governmental quarter and their relatives.	Occupational health services, with specialist support from national health authorities and psychologists. Regular GPs to issue sick leaves or referrals to specialized mental health services if needed.	The occupational health services invited the exposed employees to a consultation including a screening assessment and at least three follow-ups after 3–4 weeks, 3–4 months and 12 months. If there was a need for referrals to specialized psychiatric services and/or sick leaves, this was generally to be issued by their regular GPs. Two factors were emphasized in the selection of this corporate model: to get back to normal early and take part in the workplace community with other colleagues present at the bombing, which aimed at their workplace.	[38]
Medium/long-term	Survivors of the Utøya youth camp attack and their families.	Municipal multidisciplinary crisis teams, designated contact persons, other primary care or specialized health personnel as outlined by the municipality.	The municipalities should proactively contact the survivors of the Utøya attack. It was recommended that each survivor was given a designated contact person in the municipality that would ensure continuity in the follow-up, which focused on stabilization, practical assistance and support. Furthermore, that the contact was frequent early on and eventually adapted to personal need. The follow-up was to be maintained at least 1 year after the attack and include screening assessments to be conducted at 5–6 weeks, 3 months and 1 year after the attack. If the contact person was not a health practitioner, he/she was to make sure that such screening was performed by a health practitioner that could refer to specialized treatment if needed. The screening instrument was developed based on experiences from school shootings, the 9/11 terrorist attacks and Hurricane Katrina. The follow-up was to be adapted to the municipality’s available resources and competence. Recommendations were also sent to schools and universities on facilitation of practical, educational and social support to youth affected by the attacks. Moreover, during the first 18 months, weekend reunions were organized for the bereaved and one-day reunions for the survivors and their families.	[38, 57, 59–62]

**Table 4:** Information identified about the psychosocial care response, Paris 13 November 2015 attacks, France. Acute = first hours/days after attacks. Medium/long-term = weeks/months/years after the attacks

Timing	Target population	Providers	Description of psychosocial care	References
Acute	Individuals who were in the unsecure areas of the attacks: 381 according to report.	The first responders, including 430 firefighters from the Paris Fire Brigade, civil security associations (e.g., the Civil Protection, Red Cross, Order of Malta), the police, gendarmerie (military police) and the SAMU (emergency medical services/paramedics).	The first responders provided psychosocial support to non-injured persons in the areas of the attacks and collected information about their identity, as far as possible.	[63]
Acute/ medium	Survivors, their families, the bereaved, witnesses and others affected by the attacks.	Emergency psychosocial support units (CUMPs) from Paris and other departments in France. There is a national network of CUMPs. Every department has a CUMP organized by the regional health agency and connected to the SAMU. They are composed of voluntary health professionals such as psychiatrists, psychologists and nurses trained to provide early psychosocial care in crisis. The CUMPs are headed by a psychiatrist.	CUMPs conduct defusing to alleviate acute stress and standardized assessments to assess the risk of future posttraumatic stress reactions. They usually intervene only during the first month, and provide information about access to healthcare after the acute phase. They may assist in accessing appropriate follow-up with, e.g., GPs or psychiatrists in order to prevent PTSD and other mental health disorders. On 14 November 2015, the Health Emergency Medical Centre coordinated the organization of psychological support. CUMPs were established in two Parisian town halls, at the Military School and the Legal and Forensic Medicine Institute. The city of Paris held information campaigns, and people affected by the attacks could come spontaneously to receive consultations. In the first 20 days after the attacks, 316 practitioners from the CUMPs intervened, i.e., approximately 1/5 of the CUMP practitioners that could potentially be mobilized in France.	[63–67]
Acute	646 persons impacted by the attacks: 424 directly involved (injured, life threatened or in contact with dead victims), and 222 bereaved or indirectly affected by the attacks.	Psychiatrists, psychologists and CUMP practitioners at a provisory psychological care set-up at the Hotel-Dieu Hospital in the centre of Paris, organized in response to the terrorist attacks. Collaboration with emergency doctors at the hospital.	Psychological care was typically provided by pairs of psychiatrists and psychologists at the hospital. The psychological care set-up was located next to somatic services which could facilitate the provision of psychological care in addition to treatment of injuries. There was also a forensic unit in this hospital where physical and mental health consequences of the attacks could be recorded for legal purposes. This post-attack psychological care set-up remained open approximately 4 weeks after the attacks.	[67]
Long-term	Survivors and the bereaved from the attacks.	CUMP practitioners.	At the anniversary of the attacks and the re-opening of the Bataclan theatre 1 year after the attacks, CUMP practitioners were present to provide psychosocial care if needed.	[68]
Long- term	Victims of the attacks.	French Victim support associations that were members of the French Victim Support and Mediation Institute (“l’Institut National d’Aide Aux Victimes et de Médiation (INAVEM)”, today named France Victimes).	The French Victim support associations that were members of the French Victim Support and Mediation Institute offered free psychological support to the victims. They additionally offered consultations for legal or social support.	[69, 70]
Long- term	Those directly exposed to the attacks with physical or psychological sequelae, the bereaved, and the relatives of injured survivors (spouse, cohabiting partner bound by civil union, ascendants and descendants up to the third degree, brothers and sisters).	Mental health practitioners in the public health services, in the private sector participating in the public health services, and in the liberal private sector (e.g., private practices, private clinics).	Patients could be referred by their general practitioners, the CUMP or associations like France Victimes and receive fully reimbursed consultations with psychiatrists and medication if needed. These consultations/medications could be fully reimbursed during 2 years, given that it was requested within 10 years after the terrorist attacks.	[70]

**Table 5:** Information identified about the psychosocial care response, Brussels 22 March 2016 attacks, Belgium. Acute = first hours/days after attacks. Medium/long-term = weeks/months/years after the attacks

Timing	Target population	Providers	Description of psychosocial care	References
Acute	Victims, their families and witnesses.	The Federal Administration for Public Health (FOD Healthcare), the centre for crisis psychology of the federal service of defense, the services of the municipalities, with assistance from the local police services, and victim support organizations. The Red Cross and companies struck by the attack (e.g., the airport) were also important in the organization and provision of psychosocial care.	In the acute phase, the psychosocial assistance network of the local municipality was called for. This network was composed of different local services and was in charge of the psychosocial care in reception centres for non-injured victims and relatives of the victims organized at the municipal level. The psychosocial assistance was categorized into basic assistance (including sheltering if needed), information, emotional and social support, practical help and healthcare in case of health problems. The federal services for public health should appoint a psychosocial manager to coordinate the psychosocial care response. In case of large-scale events, specialized assistance above local level should be provided on, e.g., collection and treatment of information (concerning victims) in a central information point, acute psychosocial care, phone lines for affected people and relatives, collaboration and information exchange with the Disaster Victim Identification team of the federal police and eventual support in the reception structures. From 2 p.m. on the day of the Brussels attacks, a reception centre for the close ones of victims was opened at a military hospital. Representatives from the medical services, the police, the defense and the legal authorities were present at the centre. During the acute phase, the main coordination of the psychosocial care was at the federal level. The Red Cross assisted with the organization. There is a psychosocial intervention plan which has two phases: an acute phase and a long-term phase. A part of this plan is that the centre for crisis psychology of the federal service of defense gives psychosocial support during crisis.	[56, 71]
Acute	General population.	Cities and municipalities.	On a local level, the cities and municipalities were responsible for providing support. This could for example be to set up a centre for first psychosocial aid, in cooperation with the police.	[56]
Long-term	Victims and families.	Community level (there are in total four, each with own government: one in Brussels, as well as a French-speaking, a Dutch-speaking and a German-speaking).	In the long-term, the responsibility for the psychosocial care after the attacks was transferred from the federal level to the communities. The public health department of the federal public services was responsible for the organization of an adequate transfer toward the local communities that were competent to ensure necessary support during the post-acute phase. A lack of long-term psychosocial follow-up was reported, due to lack of communication between the federal and the local authorities, resulting in no overlap between acute and long-term help.	[72, 73]

#### Norway

There were quite detailed documents on post-disaster psychosocial care issued from the public authorities both before and in response to the 2011 terrorist attacks in Norway (Additional file 1). The texts were often formulated as recommendations to allow for flexibility for the local municipalities to adapt the provision of psychosocial care to their available resources. A new national guideline for psychosocial interventions in the context of crises, accidents and catastrophes had been developed shortly before the 22 July 2011 terrorist attacks [54]. It was published by the Norwegian Health Directorate in the immediate aftermath of the attacks, and outlined, e.g., principles for psychosocial care, roles and responsibilities of different actors in crises and catastrophes, relevant laws, needs for training and specific sections on care for children and adolescents, asylum seekers and refugees in the context of catastrophes. It referred to international guidelines and national guidelines in some other countries, and a Norwegian translation of the European Network for Traumatic Stress (TENTS) guidelines for psychosocial care following disasters was included as an Additional file 1. After the attacks, specific recommendations were made concerning the content, target populations and providers of the psychosocial care response to the attacks [38]. They differed in some aspects to the national guideline developed prior to the attacks. Whereas the national guideline favored a principle of watchful waiting, the post-attack response included proactive follow-up of two target populations [38, 54, 57]. The psychosocial care to other affected civilians who were not part of these target populations relied on the decision of the municipal crisis team or the urgency medical services in Oslo (Oslo Legevakt) or on self-referral.

The psychosocial care responses to the attacks included two distinctive models to anticipate the mental health risks and long-term follow-up of two highly exposed target groups (Table 3). A proactive primary care-based outreach model was outlined for the survivors of the shooting at the Utøya island youth camp and their families, whereas a corporate model effectuated by the occupational health services was outlined for the employees who were in the government quarter at the time of the bombing in Oslo [38, 57]. Both models comprised screening assessments throughout at least 1 year after the attacks. As for the pre-attack plans, the municipalities were under statutory obligation to establish emergency medical plans, including psychosocial care [74]. Primary care-based multidisciplinary crisis teams were to provide acute psychosocial care. It was up to the municipality to determine the composition of the crisis team. Among the professions frequently represented were medical doctors (typically GPs), psychiatric nurses, school nurses, social workers, psychologists, the police, and/or priests or other religious leaders [75]. Some municipalities organized multidisciplinary crisis teams together. In general, the regular GPs who were part of the municipal health services would typically coordinate the long-term follow-up after crisis. However, the proactive outreach model designed after the 2011 attacks relied on the coordination and continuity of follow-up by designated contact persons. It was up to the municipalities to designate the contact persons who usually were someone other than the GP. This choice was based on research from the 2004 tsunami catastrophe where some survivors reported unmet needs for healthcare in a follow-up that was based on GPs [76]. The Norwegian Red Cross assisted in the establishment of a national support group for and by those affected by the 22 July attacks in august 2011, around a month after the attack [38]. The support group provided peer support and worked for the rights and interests of those affected by the attacks. They were also important advisers for the Norwegian Directorate of Health in the monitoring and adaptation of long-term follow-up of those affected by the attacks [57].

#### France

The documents from the French health authorities were largely formulated as plans with quite detailed information on the organization of a national network of emergency psychosocial care units (CUMPs) before the attacks occurred. The organization of acute post-disaster psychosocial care and the training of its providers were outlined in a legal ordonnance in the public health code of the French Ministry of Health in 2014, i.e., the year preceding the Paris terrorist attacks [64], and addressed in national plans on the emergency medical responses to different types of crises, namely the “white plan” for the hospitals/health institutions and the “Orsan plan” for the health regions [65, 77]. The white plan referred to literature on experiences from prior terrorist attacks and catastrophes in France and comprised information sheets to affected individuals and screening schemes to evaluate the acute psychological reactions and need for follow-up [65]. According to a personal communication from a representative of the French ministries, the emergency medical response to terrorist attacks was additionally addressed in a confidential part of the security plan “ORSEC”. We were therefore unable to examine if the latter also addressed the acute psychosocial care response. The actual psychosocial care response to the Paris November 2015 attacks was described in articles, yet there was limited information on the target populations for care [66, 67]. The psychosocial care responses to the November attacks in Paris were based on experiences from prior terrorist attacks in France, including a multi-site attack occurring 10 months earlier in the same area (e.g., against the Charlie Hebdo newspaper redaction) [8]. A national network of CUMPs organized by the regional health agencies (Agence régionale de Santé, ARS) under the auspices of the SAMU (emergency medical services) [64] had been developed in the aftermath of a terrorist attack in Paris in 1995 [63]. There was a CUMP in every department composed of a team of volunteer psychiatrists, psychologists, nurses and other trained personnel. They provided psychological support in the immediate phase or within the first month as well as information about access to other healthcare alternatives in the longer term. The CUMPs also issue medical certificates for psychological trauma [65]. Due to the national network, several CUMPs could be activated in case of a mass casualty incident. Moreover, there was a national framework for training of CUMP practitioners [78]. After the attacks in November 2015, several CUMPs from Paris as well as other departments in France provided care in different locations in Paris (Table 4) [66]. Even if the CUMPs usually intervene only during the first month, they continued the follow-up for over a month in some cases after the November attacks [63]. Yet, we found no mention of any systematic long-term follow-up or screening assessments beyond a month. In addition to the acute psychosocial care provided by the CUMPs, a provisory psychosocial care unit was organized at a hospital in the city center and remained open the first month after the November attacks [67, 79]. This hospital unit had been established in the wake of the January 2015 attacks in Paris. However, the hospital-based psychosocial care unit remained open longer after the November attacks compared to the January attacks. In the long-term, the victim support associations that were members of the French Victim Support and Mediation Institute (“l’Institut National d’Aide Aux Victimes et de Médiation (INAVEM)”, today named France Victimes) offered free psychological consultations to victims of the terrorist attacks [69, 70]. In 2016, following the Paris attacks, the new information system “SI-VIC” was developed to consolidate a single list of victims after terrorist attacks and other mass casualty incidents in order to facilitate the support of victims and make contact with their relatives as well as to visualize the impact of the event on the provision of care (number of hospital beds available) [80]. SI-VIC was initiated by the French Health Ministry and managed by the Digital Health Agency. The SI-VIC system has an inter-ministerial function, integrating administrative procedures and the formal recognition of being a victim in addition to facilitating the delivery of healthcare.

#### Belgium

The federal public health services in Belgium updated their psychosocial intervention plan approximately 2 months before the 22 March 2016 Brussels attacks [56]. This plan primarily focused on the set-up of reception centres and telephone lines in the acute phase and did not refer to international guidelines or research. We found little documentation of the psychosocial care provided in response to the attacks or its providers and target populations. However, a report from a Parliament audition 10 months after the Brussels attacks described that it was difficult to obtain sufficient information and psychosocial care, and that the roles and responsibilities of different actors in psychosocial care should be clarified [73]. Furthermore, an ensuing report published in 2018 highlighted several recommendations for future psychosocial care, such as educating more professionals in psychotraumatology, establishing an expert centre and ensuring proactivity and continuity in the psychosocial care [72]. This report also described different providers of psychosocial care and support services, and referred to international guidelines. Hence, possible shortcomings in the pre-attack psychosocial care preparedness seem to have been emphasized in the health authorities’ publications after the attack.

In the acute aftermath of the Brussels attacks, the local municipalities (communes) were in charge of setting up reception centres, including the possibility to stay overnight, while the federal services were responsible for the coordination of psychosocial care (Table 5) [56]. Every municipality was responsible for integrating a psychosocial intervention plan in their general crisis plans. This comprises, e.g., the establishment of a psychosocial intervention plan network, the provision of places appropriate for providing care to affected individuals and their close ones, organizing the provision of information to the general population and those affected, planning transportation options towards reception centres and establishing agreements to resolve potential needs for meals, clothing, translators, medications, etc. The provinces and municipalities were also responsible for organizing trainings. The psychosocial assistance network of the local municipality, which was composed of different unspecified local services, was in charge of the psychosocial care in the reception centres. In case of large-scale events, specialized assistance above local level should also be provided concerning, e.g., the collection and treatment of information on victims in a central information point, acute psychosocial care, phone lines for affected people and their relatives, collaboration and information exchange with the Disaster Victim Identification team of the federal police and eventual support in the reception structures. A psychosocial manager should be appointed to coordinate the psychosocial care response, while the Federal Inspector of Hygiene was responsible for the overall crisis coordination on behalf of the minister in charge of public health. The public health department of the federal public services was responsible for the organization of an adequate transfer toward the local communities to ensure necessary support during the post-acute phase.

On the day of the Brussels attacks, a reception centre for the ones close to the victims was opened at a military hospital [71]. Representatives from the medical services, the police, the defense and the legal authorities were present at the centre. In the long term, the responsibility for the provision of psychosocial care was transferred to the community level [56]. The reports of a lack of follow-up in the longer-term promoted the development and recognition of victim support associations [72, 73, 81].

In all three countries, telephone helplines were available and accessible for the general population, and NGOs such as the Red Cross contributed in the psychosocial care response.

## Discussion

Before the terrorist attacks under study occurred, all three countries had national plans or guidelines for the provision of post-disaster psychosocial care. In the immediate aftermath of the attacks, reception centres to provide acute psychosocial care were set up in all countries. Yet, the psychosocial care responses differed in terms of organization and content, particularly in the long-term. Furthermore, the availability and levels of details in the national plans, guidelines and other documentation of the psychosocial care responses varied between countries. The documents from the Norwegian Health Directorate were largely formulated as guidelines and recommendations and included particular descriptions of potentially vulnerable groups such as children and refugees. The documents from the French and Belgian health authorities were formulated as plans rather than guidelines or recommendations. Characteristics of the attacks, the exposed populations as well as the health system and other support systems of the country where they occur may be relevant to take into account when planning and implementing a psychosocial care response.

### Psychosocial care responses according to characteristics of the attacks

This study covered three large-scale, multi-site terrorist attacks. There were differences between the attacks which may possibly have impacted the psychosocial care responses.

The study demonstrates that the geographical context is of importance for the psychosocial care response. Firstly, the resources and availability of professionals trained for providing psychosocial care may vary between urban and rural locations. Secondly, the geographical context may influence the facility of reaching and identifying exposed individuals. All the attacks under study struck the capital of their country, but the 2011 Norway attacks also involved a shooting spree on the small Utøya island in a rural municipality. The geographical limitation of the island made it possible to identify all those who had been directly exposed to the shooting spree on Utøya. This may have facilitated the implementation of a proactive outreach model. In France, the November 2015 attacks took place in various crowded places in the Paris urban area (suicide bombings outside football stadium, shootings at several cafes and restaurants and at a concert theater). In this context, survivors who were not seriously injured may have fled the scenes of the attacks before rescue personnel could identify them and offer psychosocial care. Consequently, access to care may for many have depended on self-referral. In this setting, information campaigns on the psychosocial care offers may be particularly important to reach individuals with or at risk of developing health problems after the attacks. This may also have been the case for the two attack sites in Brussels, Belgium (airport and metro station) and for the bombing in Oslo, Norway. Notwithstanding, the bombing in Oslo occurred in the government quarter in the midst of the summer vacation period. Those who were at work in the affected ministries were easily identified. Therefore, it may have been more difficult to identify all the exposed individuals after the attacks in Paris and Brussel.

Moreover, previous experiences impact the psychosocial care response. Paris was struck by terrorist attacks in January and November within the same year. Some neighborhoods were heavily affected by both attacks. Consequently, the emergency psychosocial units had recent experiences with providing care in this area upon which they could draw valuable experiences from in the psychosocial care response to the November attacks [66, 67]. In contrast to France, Norway had no recent experiences with terrorist attacks. This may have contributed to the fact that new models for follow-up were developed in response to the attacks in Norway, while France applied emergency psychosocial care units that had been developed in response to and activated after previous terrorist attacks. This was also reflected in the fact that terrorist attacks were addressed more specifically in the documents on psychosocial care responses in France also before the attacks, while in Norway and Belgium there was a broader focus on crises and catastrophes in general.

A particular feature of the 22 July Norway attacks in 2011 was that the terrorist survived and had a trial against him beginning 9 months after the attacks, lasting approximately 2 months [40]. The trial may have been a potent reminder of the attacks and possibly highly stressful for the survivors as many of them also testified. It may thus have been particularly beneficious that the proactive outreach was to be pursued for at least a year, i.e., throughout the end of the trial, with a designated contact person to ensure continuity in the follow-up.

### Psychosocial care responses according to characteristics of the exposed populations

The psychosocial care response depends on characteristics of those who were exposed. In the Brussels attacks, two of the suicide bombings occurred at an international airport [35]. A significant number of those affected lived and potentially needed follow-up in different countries. The majority (18 of 32) of the deceased were of non-Belgian nationality [35]. In the November 2015 Paris attacks, there were 24 persons of non-French nationality who were killed [34]. This may have been a challenge for the provision of psychosocial care, as the long-term follow-up must take place in different countries. In these circumstances, collaboration across borders is essential to coordinate acute and long-term follow-up of terror-exposed survivors and bereaved who are residents in other nations.

In Norway, there were many adolescents killed during the attacks, and many survivors were adolescents or young adults [36]. The young age of those concerned was one of the factors that favored the development of a proactive outreach model [57]. After the attack, the survivors and the bereaved from the Utøya attack were geographically dispersed in rural and urban municipalities across the entire country. Therefore, the long-term follow-up involved a large number of municipalities with different resources available. Since many survivors were adolescents and young adults on the brink of moving away from their family to begin their studies some weeks after the attack, the responsibility of the follow-up could change between different municipalities. The schools and educational institutions may be important in the long-term follow-up of youth, both to identify those in need of help and to provide psychosocial and educational support [9]. Indeed, recommendations on facilitating the return to school through practical and psychosocial support measures were sent to the schools after the Norway attacks [59, 60]. Even if children and adolescents were not directly exposed to the attacks in Paris and Brussels as in the Norway attacks, children may still have been highly affected as family members of victims or through media exposure. An article on the acute psychosocial support to children after the attack in Nice on July 14 2016 highlighted that up until then there was no pediatric component in the French emergency psychosocial units (CUMPs) [42]. The symptoms of stress and psychological suffering in children often differ from those in adults [82]. Their symptoms may also more easily be overlooked as children might not access health services by their own initiative but rely on their caregivers to access healthcare. Indeed, a study after the 2001 World Trade Center attacks in the US found significant levels of unmet needs among children in New York [14]. It is therefore important to develop public health strategies to identify and cover the needs for psychosocial care in children.

A key objective of psychosocial care after terrorist attacks is to identify persons at risk and prevent that they develop PTSD or other long-term health problems. Two fundamental questions are how to identify persons at risk and how to follow them up. Proactive outreach and active monitoring depend on the definition of a target population: who should be included in potential screening assessments? With respect to the attacks in Paris and Brussels, we did not find any recommendations of active monitoring with systematic screening assessments in the long-term aftermath of the attacks. Even if there were two target populations that were quite easily defined after the 2011 Norway attacks, one could, as previously mentioned, also have identified individuals at risk of developing PTSD or other health problems that could be eligible for inclusion in systematic screening programs based on the acute psychosocial care provided after the attacks in France and Belgium. Screen and treat approaches have been recommended after disasters, as survivors with mental health problems are often not detected through regular healthcare pathways [83]. Yet, problems with registration of victims or data sharing may be an obstacle to implement screening assessments [27]. As for the attacks in Norway, the geographical limitation of the Utøya island where the shooting spree took place and the shared affiliation to a workplace community at the site of the bombing in the government quarter in Oslo, may have facilitated the identification of individuals at risk of posttraumatic health problems and the implementation of long-term screening assessments. Nonetheless, a follow-up with screening assessments beyond the acute phase has also been implemented after terrorist attacks at metro stations and concerts arenas, such as the London 2005 bombings, the Manchester Arena 2017 bombing and the Utrecht 2019 tram shooting in the Netherlands [83–85]. The establishment of screening programs for longer-term follow-up seems thus more determined by different policies for psychosocial care between countries rather than characteristics of the attacks. Indeed, citizens in the UK who had been affected by the Paris or Brussels attacks were invited to a screen and treat program [86]. Notwithstanding, more research is needed to appraise the efficiency, advantages and disadvantages of screening programs and of other types of psychosocial care interventions.

### Psychosocial care responses according to characteristics of the health systems

One of the objectives of this study was to better understand how the psychosocial care responses may have been influenced by the nature of the health systems. The countries’ health systems differed for instance in terms of gatekeeping and the share of the population with a regular GP. In Norway, there was a principle of lowest effective care with a regular GP scheme where over 99% of the population had a regular GP that served as a gatekeeper for referrals to specialized health care [46, 54]. The fact that primary care services played a fundamental role generally in healthcare may have facilitated the organization of a primary care based follow-up also in the context of the terrorist attacks. Indeed, research indicates that the use of GPs was more common in survivors of the Utøya attack in Norway than in survivors of the 13 November attacks in France [87–89]. The psychosocial care response in Norway relied on multidisciplinary primary care services, including both health professionals and other professionals, in the local municipalities. Contrastingly, the emergency psychosocial units (CUMPs) in France were organized at a regional level and were mainly composed of practitioners in specialized mental health care, such as psychiatrists, psychologists and psychiatric nurses. Although there were not systems with full gatekeeping and therefore more direct access to specialized health services, the majority of the population had a regular GP also in France and Belgium [49, 53]. The mental health and psychosocial support guidelines published by the Inter-Agency Standing Committee (IASC), include a triangle model with four levels. The broad base of the triangle consists of “basic services and security” and narrows down towards the apex upwards via “community and family support” and “focused non-specialized support to, eventually, “specialized clinical mental healthcare”. With every step upwards in the triangle a more specialized level of care is utilized [90]. European guidelines for post-disaster psychosocial prescribe a similar “stepped care” model [22, 25, 91]. Within such a model, a regular GP, for instance, may facilitate the coordination and continuity of care, and research indicates that long-lasting patient relationships with a regular GP are associated with positive health-related outcomes [92, 93]. The regular GP may further have an overview of the affected individuals’ prior health problems and social situation, which may be valuable in post-disaster follow-up. Due to a high workload and workforce pressures, it may nonetheless be challenging to organize a proactive post-disaster follow-up with the GPs [76]. We have not found data on the use of different types of healthcare in the civilian survivors of the Brussels attacks in 2016. There are multidisciplinary crisis teams in Belgium, but we do not know if or to what extent they intervened in the aftermath of the attacks [94]. However, there were organizational factors with potential implications for the post-attack psychosocial care. There were reports of a lack of follow-up in the longer-term after the Brussel attacks [72, 95]. This might have been a failure related to the split responsibility between the federal and community-level services. The federal services were responsible for the coordination of the psychosocial care in the acute aftermath, and next the responsibility for the provision of psychosocial care was transferred to the community level in the long-term [56]. This study covered three high income countries with a relatively high degree of publicly funded healthcare. Even if these countries may be better equipped to provide health services to their citizens than low income countries, the delivery of psychosocial care after terrorist attacks and similar mass casualty incidents remains complex and challenging [96, 97].

It was beyond the scope of this study to analyze the roles of the victim support organizations in the provision of psychosocial care in different countries, yet our results indicate that victim support associations played important roles for long-term psychosocial care in all countries, though in somewhat different ways. In France, the victim support associations that were members of the French Victim Support and Mediation Institute offered free psychological consultations to victims of the terrorist attacks [69]. In Norway and in Belgium, support organizations for and by those affected by the terrorist attacks provided important peer support as well as initiatives to promote the rights and interests of those affected by attacks, but they did not have a responsibility for organizing healthcare services. The organization, roles and experiences of victim support organizations in different countries merit further attention in future research.

### Implications for future psychosocial care responses and research

There is still a scarcity of knowledge about the most efficient ways of organizing and providing psychosocial care in response to terrorist attacks [28]. Our study suggests that the characteristics of the attacks and the health systems are central in the shaping of the psychosocial care response. The organization and providers of psychosocial care must be adapted to the overarching health system. Different actors may be best suited to provide psychosocial care in different countries. Anyhow, it is important that policies designate relevant actors in the provision of post-disaster psychosocial care. Furthermore, that the designated providers of psychosocial care ideally receive training in advance tailored to their role and responsibilities. Notwithstanding, the heterogeneity of the psychosocial care responses to the three terrorism cases outlined in this study reflects the need of internationally recognized standards for the planning, delivery and evaluation of psychosocial care after terrorist attacks and other mass casualty events. Although European and other international guidelines for post-disaster psychosocial care exist, they lack a standard or a practical model on how to effectively register affected individuals (or combine registrations from different agencies) considered as target populations for psychosocial care and health monitoring on the short and longer term as well as a psychosocial care evaluation framework [17–20]. In a meta-analysis of two decades of post-disaster psychosocial care in the Netherlands, registration was identified as a recurring problem in the acute phase with negative consequences for the recovery phase [22]. Recently, registration problems were again found to hinder the proactive longitudinal health monitoring with psychosocial care backup organized by the local municipal health region in the 2 years after the Utrecht tram shooting on 18 March 2019 [85]. A reliable registration generated in the hours and days after the attack could provide an overview of the target population, broader than those killed and severely injured, and aid the short and long term psychosocial follow-up by health authorities, practitioners and researchers. If a practical registration standard or model is developed internationally, this could benefit the psychosocial care response to terrorism across country borders. Since terrorism is an international threat and the victims often have different nationalities, a mapping of existing structures for mental health services and psychosocial care in different countries may facilitate the coordination of the follow-up of victims when an attack strikes. This study indicates that, without harmonization of health monitoring and evaluation models, the methods and interventions applied to screen and support target groups over time and to evaluate services will differ in focus, quality and quantity. A standardized framework integrated in international psychosocial care guidelines may, when implemented nationally and locally, strengthen the public health response to terrorist attacks internationally. This study indicated that better documentation is needed about the planned as well as the actual provision of psychosocial care after terrorist attacks. The World Health Organization has emphasized the need for clear plans for psychosocial care after disasters [16]. Some of the aspects that should be covered are an identification of all relevant stakeholders and resources, validated questionnaires for needs assessments and mental health status, guidelines for care of children and a plan for assessment for psychosocial distress in the community. Monitoring of psychosocial care interventions through systematic data collection may guide the decision-making on, e.g., whether ongoing interventions should be modified and for how long they are needed. This could facilitate an ensuing evaluation of the effectiveness of specific interventions. A systematic data collection in the early, intermediate and long-term phases could further lay the foundation for research providing more generalizable knowledge. Since terrorist attacks are unforeseen events, a framework for monitoring, evaluation and research should be established in advance [90]. It could then be efficiently adapted and implemented when an attack occurs. An international discussion and agreement on a set of relevant measures on health, socioeconomic status and healthcare utilization to be assessed across countries and attacks could improve the comparability of results and strengthen our knowledge on best practices for psychosocial care responses to terrorist attacks and other mass trauma.

### Strengths and limitations

This study provides new insight into the planned and applied psychosocial care interventions after terrorist attacks in three European countries. Since there is little research on the best practices for post-disaster psychosocial care, it is especially important to synthesize grey literature on practices across countries. Information on the content, providers and rationales of psychosocial care interventions, and whether changes were made when applied after terrorist attacks, may be primarily found in grey literature [33]. The data were reviewed and discussed by a multidisciplinary team of researchers, and the analysis covered documents in the national languages (French, Dutch and Norwegian) in addition to English. This study is exploratory and may help determine future research priorities and initiate a mapping of psychosocial care responses across countries. This study does not assess the quality and efficiency of the countries’ psychosocial care responses, but it highlights the importance of gaining such insight. Several limitations apply to this study. Our analysis depends on the accuracy and accessibility of plans, guidelines, policies and other documents describing the psychosocial care responses in the different countries. The documents identified from the respective countries varied in form and content, and they did not allow for an accurate comparison of the psychosocial care responses between countries. Therefore, it was not evident to define which documents to include or not or to compare the psychosocial care responses. It cannot be guaranteed that all relevant information has been found. We may have missed relevant documents due to confidentiality or that they were otherwise difficult to obtain. Nonetheless, we endeavored to search information in a variety of ways.

Furthermore, we aimed at gaining insight into what the national authorities envisaged and required in a psychosocial care response in their country. We therefore assessed plans, guidelines and documentation on a national level and not at lower administrative or geographical levels where there may also be important information on psychosocial care responses. The information we retrieved on psychosocial care differed between countries in terms of the level of details. The descriptions of the psychosocial care responses that we identified were most detailed for the 2011 Norway attacks and least detailed for the 2016 Brussels attacks. It is important to underscore that detailed descriptions do not necessarily mean that the actual care provided was sufficient. Similarly, a lack of detailed descriptions does not necessarily indicate that there was a lack of care. The content and quality of the plans do not necessarily reflect the content and quality of the psychosocial care actually provided. We assessed the plans and other relevant literature on psychosocial care approximately five (Belgium), six (France) and ten (Norway) years after the attacks under study. The time difference might have influenced the availability of literature. Grey literature is often not available in academic databases and might eventually be removed from the web sites of official bodies. It may therefore become more difficult to retrieve as time passes, e.g., as new guidelines or plans are developed. Moreover, recommendations for psychosocial care may change over time; experiences from earlier attacks may have influenced those occurring later. We included one case of terrorism from each country. The responses to the attacks under study may not be representative for the psychosocial care provided after other terrorist attacks or other mass trauma neither in the same country nor in other countries. Furthermore, the data we retrieved on the percentage of the population who had a regular GP were not necessarily comparable. In Norway, the percentage was based on register data, whereas it was based on surveys with self-reported and potentially more inaccurate data in France and Belgium. The latter dated from 2007 and 2008, respectively, and the situation might have changed since. More recent numbers from 2019 indicated that 9.9% of the population in France did not have a regular GP or other regular physician [98]. We have not succeeded in finding more recent data on the percentage of the population with a regular GP in Belgium, however, it has been reported that 82% of the insured population in Belgium had contact with a GP in 2017 [50]. It is important to underscore that this study did not assess the quality of different types of health systems. Health systems are complex and it was beyond the scope of our study to do a complete assessment of the available psychosocial services in each country. More comprehensive data on this are available elsewhere [47, 48, 94, 99, 100].

Finally, this study covered three Western European countries with relatively accessible and well developed healthcare. Although there were differences between the countries under study, the organization, availability and documentation of psychosocial care responses to terrorist attacks may differ further with respect to countries with less developed and less accessible healthcare.

## Conclusion

Despite the existence of international guidelines on post-disaster psychosocial care, there were important differences between the three studied countries in the psychosocial care responses to large-scale terrorist attacks. In order to build better practices, a mapping of the content and organization of post-disaster psychosocial care in different countries should be established as well as a cross-country framework for monitoring and evaluation research. It is essential to gain knowledge across national borders on the quality and efficiency of different psychosocial care responses to strengthen our preparedness for terrorist attacks and similar mass casualty incidents internationally.

## Supplementary Information

**Additional file 1.** List of the reviewed documents and web sites concerning the psychosocial care responses.

## Data Availability

The data material reviewed in this article is listed in the additional file 1.
