# Supplementary material for: Psychosocial care responses to terrorist attacks: a country case study of Norway, France and Belgium
Source: BMC Health Serv Res. 2022 Mar 24;22:390. doi: 10.1186/s12913-022-07691-2 (PMC8953389; doi:10.1186/s12913-022-07691-2)
Supplement: Supplementary file 1 — Additional file 1. List of the reviewed documents and web sites concerning the psychosocial care responses. [file 12913_2022_7691_MOESM1_ESM.docx]

**Additional file 1:**

**List of the reviewed documents and web sites concerning the psychosocial care responses**

**Norway**

Helse- og omsorgsdepartementet (2007). Overordnet Nasjonal Helse og sosialberedskapsplan. Available from:

<http://www.regjeringen.no/upload/HOD/Vedlegg/Planer/HelsesosialberedskapsplanNY.pdf>

Helse- og omsorgsdepartementet (2008). Stortingsmelding 47: 2008-2009 Samhandlingsreformen Rett behandling – på rett sted – til rett tid. Available from:

www.regjeringen.no/nb/dep/hod/dok/regpubl/stmeld/2008-2009/stmeld-nr-47-2008- 2009-.html?id=567201

Helsedirektoratet (2011). Veileder for psykososiale tiltak ved kriser, ulykker og katastrofer. IS-1810.

Helsedirektoratet (2011). Letter from the Norwegian Health Directorate to the regional health authorities and the municipalities, including appendices with screening assessment schemes and guideline for the screening assessments. 11/5231.

Helsedirektoratet (2011). Helsemessig og psykososial oppfølging av rammede etter 22.07.11. Oppsummering av spørreskjema til kommunene. IS-1946.

Helsedirektoratet (2012). Kartlegging av kommunenes oppfølging av de rammede etter 22.7.11. Spørreskjema til kommunene og fylkesmennene. IS-2010.

Oppfølging av berørte og rammede etter terrorhandlingene i Oslo og på Utøya 22.07.2011- tillegg til veileder (2011).

Justis- og beredskapsdepartementet (2011 - 2012). Stortingsmelding 29: Samfunnssikkerhet. Available from: <https://www.regjeringen.no/no/dokumenter/meld-st-29-20112012/id685578/?ch=1>

Helsedirektoratet (2012). Læring for bedre beredskap – Helseinnsatsen etter terrorhendelsen 22. juli 2011. IS-1984. Available from:

https://www.helsedirektoratet.no/rapporter/laering-for-bedre-beredskap-helseinnsatsen-etter-terrorhendelsene-22.juli-2011/L%C3%A6ring%20for%20bedre%20beredskap%20-%20Helseinnsatsen%20etter%20terrorhendelsene%2022.%20juli%202011.pdf/_/attachment/inline/4a3c5416-2e7f-4cee-8851-0e4a2b292460:77294d235a67e9b4291de830ea7007b884c3ed31/L%C3%A6ring%20for%20bedre%20beredskap%20-%20Helseinnsatsen%20etter%20terrorhendelsene%2022.%20juli%202011.pdf

Helsedirektoratet. Agenda kaupang (2016). Evaluering av modellen for den psykososiale oppfølgingen etter 22. juli 2011. R9154. Available from:

<https://www.helsedirektoratet.no/rapporter/evaluering-av-den-psykososiale-modellen-for-den-psykosiale-oppfolgingen-etter-22-juli-2011/Evaluering%20av%20den%20psykososiale%20modellen%20for%20den%20psykosiale%20oppf%C3%B8lgingen%20etter%2022%20juli%202011.pdf/_/attachment/inline/e958a027-6e6e-4618-a737-75557eddf887:7330fe183f4fbb4d0ea1f0f0621eeeac7544e3bb/Evaluering%20av%20den%20psykososiale%20modellen%20for%20den%20psykosiale%20oppf%C3%B8lgingen%20etter%2022%20juli%202011.pdf>

Helsedirektoratet (2014) Oppfølging av tiltak etter terroranslaget 22. juli 2011-interimsrapport pr 1. juli 2014. 12/8723-18. Available from:

<https://docplayer.me/1268560-Oppfolging-av-tiltak-etter-terroranslaget-22-juli-2011-interimsrapport-pr-1-juli-2014.html>

Helsedirektoratet (2016). Mestring, samhørighet og håp. Veileder for psykososiale tiltak ved kriser, ulykker og katastrofer. IS-2428. Available from:

<https://www.helsedirektoratet.no/veiledere/psykososiale-tiltak-ved-kriser-ulykker-og-katastrofer/Psykososiale%20tiltak%20ved%20kriser,%20ulykker%20og%20katastrofer%20%E2%80%93%20Veileder.pdf/_/attachment/inline/9170958a-0200-4f74-a842-ce505e8dbbe6:3a3fb52fc12bffd1b64e3a1567333d5d239a2167/Psykososiale%20tiltak%20ved%20kriser,%20ulykker%20og%20katastrofer%20%E2%80%93%20Veileder.pdf>

Helse- og omsorgsdepartementet (2018). Å verne om liv og helse. Nasjonal helseberedskapsplan. Available from:

<https://www.regjeringen.no/globalassets/departementene/hod/fellesdok/planer/helseberedskapsplan_010118.pdf>

Helsedirektoratet (2015). Oppfølging av erfaringer etter 22. juli 2011. Sluttrapport for prosjektperioden. IS-2384.

Kärki FU. Norway's 2011 Terror Attacks: Alleviating National Trauma With a Large-Scale Proactive Intervention Model. Psychiatr Serv. 2015 Sep;66(9):910-2. doi: 10.1176/appi.ps.201500050. Epub 2015 Jun 1. PMID: 26030322. Available from : <https://ps.psychiatryonline.org/doi/full/10.1176/appi.ps.201500050>

**France**

Arrêté du 24 février 2014 relatif aux modalités d’intervention des cellules d’urgence médico-psychologique et aux conditions de participation des personnels et des professionnels à une cellule d’urgence médico-psychologique. Available from: <https://www.legifrance.gouv.fr/jorf/id/JORFTEXT000028680791>

Arrêté du 27 décembre 2016 fixant les modalités de l’organisation de l’urgence médico-psychologique. Available from: <https://www.legifrance.gouv.fr/jo_pdf.do?id=JORFTEXT000033825631>

Direction générale de la santé, Direction générale de l’offre de soins & La Ministre des affaires sociales et de la santé (2017). Instruction N° DGS/VSS2/2017/7 du 6 janvier 2017 relative à l’organisation de la prise en charge de l’urgence médico-psychologique. Available from: <http://circulaire.legifrance.gouv.fr/pdf/2017/01/cir_41730.pdf>

Ministère des Affaires sociales, de la Santé et des Droits des femmes & Direction générale de la santé (DGS) (2014). Aide à l’organisation de l’offre de soins en situations sanitaires exceptionnelles. Available from : <https://solidarites-sante.gouv.fr/IMG/pdf/organisation_offre_de_soins_ok_bd.pdf>

Ministre de la santé et solidarités (2006). Plan blanc et gestion de crise. Guide d’aide à l’élaboration des plans blancs élargis et des plans blancs des établissements de santé. Available from: <https://solidarites-sante.gouv.fr/IMG/pdf/plan_blanc2006-2.pdf>

Direction générale de la Santé (DGS) (2+18). Agressions collectives par armes de guerre : Conduites à tenir pour les professionnels de santé. Available from : <https://solidarites-sante.gouv.fr/IMG/pdf/Agressions-collectives.pdf> Comment : Only the text specifically addressing the 13 November 2015 attacks in Paris in chapter 15 was reviewed in our study.

Rerbal, D., Prieto, N., Vauz, J., Gloaguen, A., Desclefs J.P., Dahan, B., Cesareo, E., Duchenne, J. Organisation et modalités d’intervention des Cellules d’Urgence Médicopsychologique. Recommandations de la Société française de médecine d’urgence (SFMU) en collaboration avec l’Association de formation et de recherche des cellules d’urgence médicopsychologique ‑ Société française de psychotraumatologie (AFORCUMP‑SFP). Ann. Fr. Med. Urgence (2017) 7:410-424. DOI 10.1007/s13341-017-0804-7. Available from: <https://afmu.revuesonline.com/articles/lvafmu/pdf/2017/06/lvafmu76p410.pdf>

Convention. Cellules d’urgence médico-psychologique. Available from: <https://www.aforcump-sfp.org/site/file/source/documents_utiles/cump_convention.pdf>

Premier ministre & Secrétariat Général de la Défense et de la Sécurité Nationale (2016). TACKLING TERRORISM TOGETHER. VIGILANCE, PREVENTION, AND PROTECTION AGAINST THE TERRORIST THREAT. Available from: <https://www.gouvernement.fr/sites/default/files/locale/piece-jointe/2017/08/vigipirate-anglais-v2017.pdf>

Ministere du travail, de l’emploi et de la santé (2011). Chapter PRÉPARATION ET GESTION DES ALERTES SANITAIRES in Livre des plans de santé publique. Available from:

<https://solidarites-sante.gouv.fr/IMG/pdf/LDP_2011_BD.pdf>

Ministère de la Justice & Inter-ministerial committee on victim assistance (n/a). INTER-MINISTERIAL PLAN FOR VICTIM ASSISTANCE. Available from: <http://www.justice.gouv.fr/art_pix/plan_annuel_diav_en.pdf>

Assemblée Nationale. Commission d’enquête de l’Assemblée nationale (2016). Moyens mis en oeuvre par l’Etat pour lutter contre le terrorisme depuis le 7 janvier 2015. Report No.: 3922.

Available from: <http://www.assemblee-nationale.fr/14/pdf/rap-enq/r3922-t1.pdf>

Gouvernement. Guichet unique d’information et de déclaration pour les victimes. Available from: <https://www.gouvernement.fr/guide-victimes>

Dantchev, N., Ben Younes, S., Mullner, J. *et al.* Retour d’expérience des attentats du 13 novembre 2015. Prise en charge psychologique hospitalière des impliqués. *Ann. Fr. Med. Urgence* **6,**55–61 (2016). [Available from: https://doi.org/10.1007/s13341-016-0604-5](https://doi.org/10.1007/s13341-016-0604-5)

Philippe, JM., Brahic, O., Carli, P. *et al.* French Ministry of Health’s response to Paris attacks of 13 November 2015. *Crit Care* **20,**85 (2016). Available from: <https://doi.org/10.1186/s13054-016-1259-8>

Rudetzki, F. POUR UN CENTRE DE RESSOURCES ET DE RESILIENCE : REPARER ET PRENDRE SOIN DE LA VIE. Rapport déposé en décembre 2016, puis remis officiellement à Monsieur le Président de la République et à Madame la Secrétaire d’Etat auprès du Premier ministre, chargée de l’aide aux victimes, le 8 février 2017. Available from: <https://www.gouvernement.fr/sites/default/files/contenu/piece-jointe/2017/03/rapport_de_francoise_rudetzki_pour_un_centre_de_ressources_et_de_resilience-reparer_et_prendre_soin_de_la_vie.pdf>

**Belgium**

FOD Volksgezondheid, veiligheid van de voedselketen en milieu (2018). Technische werkgroep: Psychosociale opvolging: VOOR EEN GEÏNTEGREERDE PSYCHOSOCIALE OPVOLGING VAN GETROFFENEN VAN COLLECTIEVE NOODSITUATIES. Available from: https://healthpr.belgium.be/sites/default/files/uploads/fields/fpshealth_theme_file/visietekst-printversie.pdf

V-Europe (2017). Aanslagen 22 maart onderzoekscommissie pleit voor individuele begeleiding snelle erkenning en steun voor slachtoffers. Available from: <https://www.knack.be/nieuws/belgie/aanslagen-22-maart-onderzoekscommissie-pleit-voor-individuele-begeleiding-snelle-erkenning-en-steun-voor-slachtoffers/article-normal-847639.html?cookie_check=1626280519>

BELGISCHE KAMER VAN VOLKSVERTEGENWOORDIGERS (2016). PARLEMENTAIR ONDERZOEK: belast met het onderzoek naar de omstandigheden die hebben geleid tot de terroristische aanslagen van 22 maart 2016 in de luchthaven Brussel-Nationaal en in het metrostation Maalbeek te Brussel, met inbegrip van de evolutie en de aanpak van de strijd tegen het radicalisme en de terroristische dreiging. DOC 54 1752/006. Available from: <https://www.dekamer.be/doc/flwb/pdf/54/1752/54k1752006.pdf#search=%22doc%2054%201752/006%20%2054%20%3Cin%3E%20keywords%22>

BELGISCHE KAMER VAN VOLKSVERTEGENWOORDIGERS (2017a). PARLEMENTAIR ONDERZOEK: belast met het onderzoek naar de omstandigheden die hebben geleid tot de terroristische aanslagen van 22 maart 2016 in de luchthaven Brussel-Nationaal en in het metrostation Maalbeek te Brussel, met inbegrip van de evolutie en de aanpak van de strijd tegen het radicalisme en de terroristische dreiging. DOC 54 1752/007. Available from: [https://www.dekamer.be/doc/flwb/pdf/54/1752/54k1752007.pdf#search=%22doc%2054%201752/007%20%2054%20%3Cin%3E%20keywords%22](https://www.dekamer.be/doc/flwb/pdf/54/1752/54k1752007.pdf#search=%22doc%2054%201752/007%20%2054%20%3Cin%3E%20keywords%22)

Department of the House of Representatives (2018). Investigation committee terrorist attacks 22 March 2016. Summary of the activities and recommendations. Available from: <https://www.dekamer.be/kvvcr/pdf_sections/publications/attentats/Brochure_Terrorists_Attacks.pdf>

Crisiscentrum (2016). 57 slachtoffers van de aanslagen van 22 maart verblijven nog altijd in Belgische ziekenhuizen. Available from: <https://crisiscentrum.be/nl/news/crisisbeheer/57-slachtoffers-van-de-aanslagen-van-22-maart-verblijven-nog-altijd-belgische-ziek>

Crisiscentrum (2016). 18u00 - Aanslagen : prioriteit aan slachtoffers en verwanten. Available from: <https://crisiscentrum.be/nl/news/crisisbeheer/18u00-aanslagen-prioriteit-aan-slachtoffers-en-verwanten>

Terrorisme: Nationaal noodplan (2017). Het Crisiscentrum kreeg bijkomende opdrachten toevertrouwd door het nationaal noodplan inzake terrorisme. Available from: <https://2016.ibz.be/nl/2017/05/05/terrorisme-plan-durgence-national/>

Departement Welzijn Volksgezondheid en Gezin (2016). Hulp aan getroffenen van de aanslagen blijft doorlopen. Available from: <https://www.departementwvg.be/nieuws/hulp-aan-getroffenen-van-de-aanslagen-blijft-doorlopen>

Vlaams Parlement (2017a). SCHRIFTELIJKE VRAAG: Nabestaanden slachtoffers aanslagen 22 maart 2016 - Afstemming slachtofferhulp en psychosociale ondersteuning. Available from: <http://docs.vlaamsparlement.be/pfile?id=1246373>

FOD Volksgezondheid, veiligheid van de voedselketen en milieu (2017a). Nieuw medisch interventieplan aangepast aan terroristische aanslagen. Available from: <https://www.health.belgium.be/nl/news/nieuw-medisch-interventieplan-aangepast-aan-terroristische-aanslagen>

FOD Volksgezondheid, veiligheid van de voedselketen en milieu (2016). Medische zorg, psychologische begeleiding en info voor slachtoffers en betrokkenen bij aanslagen. Available from: <https://www.health.belgium.be/nl/news/medische-zorg-psychologische-begeleiding-en-info-voor-slachtoffers-en-betrokkenen-bij-aanslagen>

Vlaams Parlement (2017b). Woordelijke verslag: Plenaire Vergadering. Available from: <http://docs.vlaamsparlement.be/pfile?id=1254442>

FOD Volksgezondheid, veiligheid van de voedselketen en milieu (2016b). Psychosociale hulpverlening. Available from: <https://www.health.belgium.be/nl/gezondheid/organisatie-van-de-gezondheidszorg/dringende-hulpverlening/psychosociale-hulpverlening>

FOD Volksgezondheid, veiligheid van de voedselketen en milieu (2016). Psychosociaal interventieplan (PSIP). Available from: <https://www.health.belgium.be/sites/default/files/uploads/fields/fpshealth_theme_file/psip_2017.pdf>

Rode Kruis (2016) Strategie 2020: Iedereen helpt. Available from: <https://www.rodekruis.be/storage/main/strategie-2020.pdf>

Rode Kruis Vlaanderen (2016). Terrorisme en dan verder. Available from: <https://www.rodekruis.be/storage/main/rodekruis-vlaanderen-terrorismeendanverder.pdf>

Service Public Fédéral (2016). Terrorisme en dan verder. Available from : <https://centredecrise.be/sites/default/files/brochure_nl.pdf>

Tele-Onthaal (2016). Federatie Tele-Onthaaldiensten in Vlaanderen: Jaarverslag 2016. Available from: <https://www.tele-onthaal.be/assets/documents/downloads/publicaties/jaarverslag/Tele-Onthaal-definitief-jaarverslag.pdf>

Federale Pensioendienst (2019). Slachtoffers terreurdaden. Available from: <https://www.sfpd.fgov.be/nl/recht-op-pensioen/burgerslachtoffers>

Awel (2018). Identiteit in de aanslag: Awel onderzoeksrapport. Vlaanderen (publisher), Brussel.

United Nations Regional Information Centre for Western Europe (2018). UN expert encourages Belgium’s balanced approach but urges action on victims of terrorism and prisons. <https://www.ohchr.org/EN/NewsEvents/Pages/DisplayNews.aspx?NewsID=23165&LangID=E>

Departement Justitie (2019). Protocol tot regeling van de werkzaamheden van het centraal loket voor slachtoffers van terroristische aanslagen en grote rampen. Available from: <https://www.om-mp.be/sites/default/files/u147/col_21-2020-bijlage_1-protocol_centraal_loket_slachtoffers_nl.pdf>

Rijksdienst voor Ziekte- en Invaliditeitsverzekering (National Institute for Health and Disability Insurance). Available from: <https://www.riziv.fgov.be/nl/Paginas/default.aspx>
